# Homeostatic-related peptides injected into the rat nucleus accumbens alter palatable eating and impact the binge-like intake of a sweetened fat diet during simultaneous μ-opioid receptor stimulation

**DOI:** 10.3389/fnins.2025.1614819

**Published:** 2025-07-14

**Authors:** Wayne E. Pratt, Cardinal Do, Alexandra M. Groome, Alana J. Smith, Allison C. Siegfried, Camryn J. Calafiore

**Affiliations:** Department of Psychology, Wake Forest University, Winston-Salem, NC, United States

**Keywords:** motivation, eating, food intake, palatable eating, hedonic eating, nucleus accumbens, peptides, opioids

## Abstract

**Background:**

The nucleus accumbens is central for directing motivated behavior and is a key node in the neural circuitry that promotes eating in response to palatable diets. We examined the impact of intra-accumbens injections of a variety of homeostatic-related peptides (HRPs) on eating elicited by a sweetened fat diet, with or without co-administration of the prophagic mu-opioid agonist [D-Ala2, N-MePhe4, Gly-ol]-enkephalin (DAMGO).

**Methods:**

Rats received surgical placement of guide cannulas above the anterior medial nucleus accumbens. Non-restricted animals were then accustomed to 2-h daily access to a sweetened fat diet. Palatable eating was examined following intra-accumbens injections of one hypothalamic HRP, alone or with co-infusions of DAMGO.

**Results:**

Nucleus accumbens injections of neuropeptide Y (NPY) and Orexin-A significantly increased palatable eating. Cocaine- and amphetamine-related transcript (CART) reduced intake. When rats received co-stimulation of μ-opioid receptors, NPY and DAMGO had a synergistic effect on food intake, whereas Orexin-A initially disrupted eating on the palatable diet and had no additive effect with DAMGO by the end of the session. Neither agouti-related protein (AGRP), melanin concentrating hormone (MCH), alpha-melanocyte stimulating hormone (αMSH), nor the stress-related peptides corticotropin-releasing factor (CRF) or urocortin impacted intake, although MCH and CRF both affected eating in response to mu-opioid receptor stimulation dependent upon the dose.

**Conclusion:**

These experiments offer insight into the regulation of hedonically motivated feeding by homeostatic- and stress-related inputs to the nucleus accumbens. This systematic examination suggests that the nucleus accumbens’ role in promoting palatable eating is not independent of the homeostatic signals that reach it from the hypothalamus and other brain regions.

## 1 Introduction

Recent changes in the global food environment have led to an increase in the prevalence of overweight and obesity over the past 50 years, leading to fundamental changes in how we think about the behavioral and physiological regulation of food-seeking and consumption. Early research into the regulation of food intake focused primarily on the homeostatic nature of eating: that is, in assessing how organisms are motivated to seek and consume sufficient energy resources in the face of energy deficit, and inversely how food consumption stops (at the end of a meal) once energy stores are repleted. Much of this research focused on identifying relevant peripheral signals of energy deficit or repletion, as well as identifying the central brain regions (such as the hindbrain and hypothalamus) that respond to those signals to activate food-seeking in times of need (Woods et al., 1998, 2004a; Havel, 2001; Williams et al., 2001; Grill and Hayes, 2012).

The rapidly increasing prevalence of obesity has often been attributed to changes in food accessibility and composition, which has led to the high availability of low-cost and calorically dense foods that were not available even 100 years ago. The homeostatic controls that served our species well when food scarcity was common are less well-adapted to the food environment that we have built for ourselves since the dawn and progression of the agricultural revolution. Palatable foods that are now easily obtainable (i.e., those that are often high in caloric elements such as fat and sugar, often combined with salt) are resistant to suppression of intake by homeostatic signaling in the brain and periphery. Exposure to these foods (as well as the weight gain that they can cause) reduces the sensitivity of brain circuitry to sense peripheral satiety signals of energy stores (e.g., insulin and leptin insensitivity; Woods et al., 2004b; Williams, 2012; Mendoza-Herrera et al., 2021) and activate plastic changes in brain regions that communicate the reinforcing and/or hedonic evaluation of food (e.g., Kelley et al., 2003; Stice and Burger, 2019). Overeating on palatable foods was adaptive in a food-scarce environment, when gaining a little weight might increase survival during the next period of scarcity. However, this same tendency has become maladaptive in our modern food environment, leading to increases in food intake, obesity, and subsequent medical complications (resulting in $260 billion in annual costs in the United States, see Cawley et al., 2021).

**FIGURE 1:**
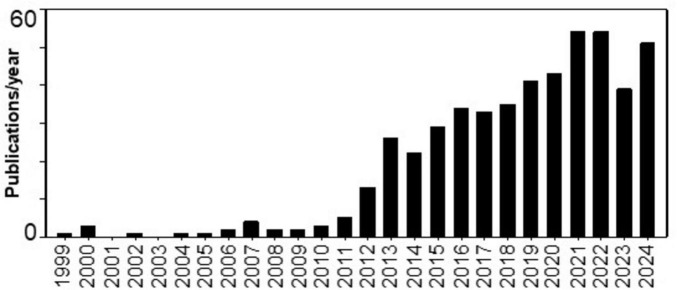
A frequency histogram of the number of citations identified by PubMed as containing the phrases “palatable feeding” or “hedonic hunger” or “hedonic feeding” or “palatable eating” or “hedonic eating” as retrieved on 8 April 2025. The first citation was found in 1999, although common use did not begin until the 2010s.

This idea that palatable foods may override homeostatic signals and be a critical component of the eating that leads to obesity is relatively new. There were early indications (in the 1980s and 1990s) of a possible role for brain “reward” systems (a term that is used broadly but also maligned, see Salamone, 2006) in modulating food intake. As one example, multiple laboratories reported that μ-opioid receptor activation within mesostriatal circuits increased eating, particularly on palatable foods (Mucha and Iversen, 1986; Bakshi and Kelley, 1993; Zhang et al., 1998; Ragnauth et al., 2000; Zhang and Kelley, 2002; Will et al., 2003; Levine and Billington, 2004; Ward et al., 2006). In some localized “hot-spots” (and notably for the purposes of this study, the anterior medial nucleus accumbens) μ-opioid stimulation was also shown to enhance the hedonic evaluation of sweet solutions (Peciña and Berridge, 2005; Smith and Berridge, 2005; Peciña et al., 2006b; Morales and Berridge, 2020). However, the rise of terms such as “palatable eating” and “hedonic hunger” did not begin to appear in the literature until the turn of the millennium, and their use has become increasingly more common from the 2010s until now (see Figure 1). Key to the development of this novel terminology may well be the simultaneously arising arguments that made a case that the intake of some types of food (particularly sweet but perhaps also fatty) may be due to similar brain mechanisms that are involved in the addictive processes underlying drugs of abuse (e.g., Colantuoni et al., 2002; Kelley and Berridge, 2002; Avena and Hoebel, 2003; Volkow and Wise, 2005; Volkow et al., 2011). It has been argued by many, including our laboratory, that although food intake is an adaptive behavior driven toward the survival of the individual and the species, some patterns of food intake may maladaptive. Various terms such as “food addiction,” “food abuse,” or “disorders of appetitive motivation” (also taking hold during the 2010s) have been used to refer to maladaptive eating behaviors (e.g., Kelley et al., 2005; Stice et al., 2013; Krupa et al., 2024), some of which may lead to obesity, and some of which may not (e.g., not all binging leads to obesity, and yet is still disruptive).

That maladaptive food-seeking and consumption may be driven by mechanisms akin to drug abuse has led many researchers to carefully examine regions of the brain that are known to be involved in drug abuse as mediators of the effects of palatable foods in overeating. A logical result of a proposed distinction between “homeostatic” versus “hedonic” eating has led many laboratories (ours included) to imply or state outright that regions such as the ventral tegmental area, the nucleus accumbens, and other interconnected forebrain areas such as the ventral pallidum are particularly important regions for “palatable” or “hedonic” eating. The temptation that results from these findings is one of a bifurcation of function, where the hypothalamus directs food seeking and intake based upon energy needs signaled by the hindbrain and periphery, while those regions of the brain that are involved in promoting food intake based upon palatability or incentives (such as the mesolimbic dopamine system and its interconnected areas) promote intake beyond that which is strictly required by homeostatic signaling of the hypothalamus.

Although it may well be that specific brain regions have specialized motivational functions, it is also the case that lumping some brain regions into a “homeostasis” camp and others into a “hedonic” camp belies the complexity of the interconnected nature of the brain. As early as the hindbrain, both sensory information that indicates food quality and homeostatic signals from vagal afferents converge, affecting food intake through interactions within the same brain circuits. Decerebrate rats, for instance, can alter their intake of offered food based on both hunger and hedonic signals (for an exceptional review, see Grill and Kaplan, 2002). In intact rats, hindbrain signals impact upstream brain regions (such as the hypothalamus and mesostriatal pathways) to promote motivated appetitive seeking behaviors, and they also work to regulate food intake once a food source is achieved. Thus, the division of different brain regions into “homeostatic” and “hedonic” regions has sparked conversation echoing the concerns of Stellar (1954) when he noted the central importance of the hypothalamus for directing motivation, but also outlined the perils of strict localization of function. As two recent examples, Liu and Kanoski (2018) made a convincing case that to separate brain regions that impact eating (such as the hypothalamus and ventral striatum) into homeostatic and non-homeostatic circuits is at best an oversimplification, and at its worst misleading. In a recent review by Woods and Begg (2016), the authors present a model in which hedonic regulation of eating overlies the same brain structures as those that control homeostatic eating. These are not the only cases that attempt to synthesize the interconnected functions of brain regions involved in directing food motivation (Kelley et al., 2005; Ferrario et al., 2016; Berthoud et al., 2017; Morales and Berridge, 2020; Marinescu and Labouesse, 2024; Stuber et al., 2025), but accounts vary from emphasizing cross-talk between homeostatic- and hedonic-related regions of the brain to an attempt to dissolve the traditional homeostatic/hedonic boundaries. With the recent success of glucagon-like peptide-1 (GLP-1) receptor agonists for promoting weight loss and suppressing food-directed motivation (e.g., Krieger et al., 2025), a more thorough understanding of how the brain modulates hedonic eating may well be relevant, as GLP-1 signaling occurs across many brain regions that are involved in promoting eating in response to both energy deficit and palatable foods (Merchenthaler et al., 1999; Cork et al., 2015).

Our laboratory has been particularly interested in the function of the nucleus accumbens in regulating food intake and food-directed motivation. Mogenson et al. (1980) first proposed an important role for the nucleus accumbens for translating “motivation to action” due to its unique positioning between classic limbic circuits (including the hippocampus and amygdala and their connections with the hypothalamus) and motor circuitry of the basal ganglia. Since that time, multiple labs have shown that the nucleus accumbens is important in directing motivation toward both natural and drug reinforcers. For instance, neuronal firing in the nucleus accumbens is impacted by intake of rewarding substances such as food and drugs of abuse (Carelli et al., 2000). Individual neurons within the nucleus accumbens predict upcoming reward based upon Pavlovian or spatial cues (Lavoie and Mizumori, 1994; Day et al., 2006; Wan and Peoples, 2006). In humans, nucleus accumbens activity not only changes in response to the presentation of palatable solutions, but its responsivity is higher in same-weight adolescents that are likely to become obese, compared to those that are less likely to gain weight in the future (Stice et al., 2011).

Using pharmacological manipulations, we and others have reported that the nucleus accumbens is involved in learning to earn food as well as to seek it out (e.g., Smith-Roe and Kelley, 2000; Pratt and Kelley, 2004; Clissold and Pratt, 2014), that it is important for promoting food intake in the presence of incentives that cue food availability (e.g., Floresco et al., 2008; Guy et al., 2011), and that distinct pharmacological manipulations within the nucleus accumbens can promote or inhibit food intake and affect the hedonic evaluation of foods (e.g., Mucha and Iversen, 1986; Ragnauth et al., 2000; MacDonald et al., 2003; Peciña and Berridge, 2005; Will et al., 2006; Pratt et al., 2009; Skelly et al., 2010; Zheng et al., 2010; Clissold et al., 2013; Pierce-Messick and Pratt, 2020). Interestingly, different neurotransmitters within the nucleus accumbens appear to affect distinct components of motivation. For instance, dopamine signaling serves an important role in energizing behavior and promoting incentive salience, while opioid signaling (particularly at the μ-opioid receptor) increases hedonic evaluation of foods and promotes intake, particularly on preferred diets (Kelley, 2004; Peciña et al., 2006b; Berridge, 2007; Salamone et al., 2007; Baldo et al., 2013). The robust eating and increased hedonic evaluations that are observed following μ-opioid receptor activation, particularly in the “hot spot” of the anterior medial nucleus accumbens (Peciña and Berridge, 2005), suggest that the nucleus accumbens is an important node for promoting eating due to the palatable properties of food.

Despite the evidence that the nucleus accumbens is clearly involved in hedonic modulation of eating, there is a relatively sparse literature examining how its function might be regulated by hypothalamic signals that arise from the arcuate nuclei or lateral hypothalamus and release HRPs into the anterior medial shell regions (for notable contributions, see Pandit et al., 2013, 2014; 2015; Liu et al., 2020). The arcuate nucleus of the hypothalamus contains two sets of neurons that are important for sensing energy signals and directing eating behavior (Woods et al., 1998). One set of neurons expresses neuropeptide Y (NPY) and agouti-related protein (AGRP), both of which promote food-seeking and consumption during energy deficit. The other set expresses cocaine- and amphetamine-related transcript (CART) and releases alpha-melanocyte stimulating hormone (αMSH), which inhibit eating in response to satiation. These two populations of arcuate neurons project to the lateral hypothalamus, activating or inhibiting (respectively) populations of neurons containing orexin/hypocretin and melanin concentrating hormone (MCH). Both orexin and MCH ultimately promote food-motivated behaviors via projections to brain regions outside of the hypothalamus. Through their projections, neurons expressing NPY, Orexin, MCH, and AgRP act in an orexigenic manner, while those that release CART and αMSH are anorexigenic. Both the arcuate nucleus and the lateral hypothalamus send projections directly to the medial nucleus accumbens shell (Sim and Joseph, 1991; Peyron et al., 1998; Kampe et al., 2009; Bharne et al., 2015; Lee and Lee, 2016), and receptors that respond to these peptides are expressed within the nucleus accumbens (often most densely in the medial shell: Widdowson, 1993; Lindblom et al., 1998; Trivedi et al., 1998; Hervieu et al., 2000; Saito et al., 2001; D’Almeida et al., 2005). This connectivity suggests that the nucleus accumbens integrates hypothalamic signaling into its direction of food motivation.

In this set of experiments, we sought to determine if individual injections of these six HRPs into the anterior medical nucleus accumbens would impact hedonically motivated eating. In individual groups of rats, we tested whether injections of a single HRP into the anterior medial nucleus accumbens affected the consumption of a sweetened fat diet offered to rats that were not food-restricted. Given prior evidence that at least some of these peptides may interact with opioid signaling to have their effects (Kotz et al., 1995; Rudski et al., 1996; Rossi et al., 1998; Clegg et al., 2002; Sweet et al., 2004; Israel et al., 2005; Furudono et al., 2006; Zheng et al., 2010), we also examined if each peptide would impact the binge-like eating observed following co-injection of the μ-opioid receptor agonist [D-Ala2, N-MePhe4, Gly-ol]-enkephalin (DAMGO) into the same region. Additionally, we examined whether the stress-related peptides corticotropin-releasing factor (CRF) or urocortin might affect palatable eating when similarly injected into the nucleus accumbens.

## 2 Materials and methods

### 2.1 Animals and housing

Male Sprague-Dawley rats (Envigo, Indianapolis, IN, USA; 9–10 weeks of age at arrival) were pair housed in clear polycarbonate cages in a temperature (∼21°C) and light controlled room with a 12-h light-dark cycle (lights on at 7 a.m.). Animals were fed *ad libitum* in their home cages with standard rat chow throughout these experiments. All procedures were conducted in accordance with NIH animal care guidelines and were reviewed and authorized by the Wake Forest University Animal Care and Use Committee. Rats were given at least 1 week to acclimate to laboratory conditions and handling prior to beginning experimental procedures.

### 2.2 Surgical procedures

Rats were anesthetized with a ketamine/xylazine cocktail (90 and 9 mg/kg, respectively). Standard aseptic surgical procedures were used to implant two stainless steel guide cannulas (23 gauge) bilaterally above the medial nucleus accumbens shell (+3.1 mm anterior and 0.7 mm lateral to bregma; 5.0 mm ventral to skull surface; nose bar at +5 mm above interaural zero). Wire stylets were placed in the cannula to maintain patency. The anterior medial region of the nucleus accumbens shell was targeted because it receives direct inputs from fibers arising from arcuate and lateral hypothalamic regions (Sim and Joseph, 1991; Peyron et al., 1998; Kampe et al., 2009; Bharne et al., 2015; Lee and Lee, 2016), and because pharmacological manipulations there have been shown to impact food-directed motivation and eating behaviors (see above). Analgesia and infection were controlled by prophylactic administration of Meloxicam (2 mg/kg, at surgery and 24-h) and Penicillin G Procaine (22,000 IU/kg, I.M.), respectively. Once surgeries were completed, animals recovered for 1 week prior to habituation to the experimental feeding chambers.

### 2.3 Feeding chambers

Clear acrylic feeding chambers were equipped with a food hopper, a water bottle, and seven infra-red eyebeams to measure locomotor behavior (Med Associates, St. Albans, VT, USA). Three eyebeams were located 5 cm above the wire floor to measure ambulation (the number of crosses from one end of the chamber to the other), and four were placed 16 cm above the floor to index rearing behavior. At one end of the chamber, the food hopper (head entry 6.4 cm above the floor) contained a high-fat, high-sucrose diet (TD.99200; Envigo; for diet composition, see Pratt et al., 2017); a water bottle was hung at the opposite end of the chamber. Med-PC software (Med Associates, St. Albans, VT, USA) continuously monitored interruptions of the IR beams and recorded the weight of the sweetened fat diet inside the food hopper at 10-s intervals. A speaker maintained an ambient level of white noise at 65 dB in the experimental room.

### 2.4 Eating paradigm and drug administration

After surgical recovery, rats were given 10 days to habituate to the feeding chambers. Each session consisted of 2-h access to the sweetened fat diet and water within the chambers. On the final 2 days of habituation, rats received mock injections to allow acclimation to microinjection procedures. On the first day, stainless steel mock injectors (30 gauge) were lowered flush to the end of the guide cannula; the second mock injection utilized cannulas that were lowered to the infusion site, 2.5 mm below the end of the guides. No solutions were delivered on mock injection days.

Experimental treatments began 48 h after the last mock injection. Each experimental group of rats were tested with multiple doses of a single homeostatic- or stress-related peptide, presented alone or with DAMGO across multiple injection days. The drugs and doses tested for each group are shown in Table 1. All drugs were prepared in a saline vehicle; each peptide and DAMGO were prepared in saline and frozen as stock solutions at −20°C. On the day of each experiment, these stocks were thawed and diluted with saline to achieve their final concentrations for injection.

**TABLE 1:** Summary of homeostatic- and stress-related peptides infused into the anterior medial nucleus accumbens during these experiments.

Peptide treatment	Source	Doses used (in 0.5 μl/side)	Doses based upon prior work by:
Neuropeptide Y (NPY)	Tocris Bioscience	0, 0.67, and 1.0 μg	Brown et al., 2000; van den Heuvel et al., 2015; Carney et al., 2023
Agouti-related protein (AGRP) (83–132) amide	Phoenix Pharmaceuticals	0, 0.235, and 0.94 μg	Pandit et al., 2015
Cocaine- and amphetamine-related transcript (CART)	Phoenix Pharmaceuticals	0, 0.01, and 1.0 μg	Yang et al., 2005
Alpha-melanocyte stimulating hormone (αMSH)	Phoenix Pharmaceuticals	0.0, 0.138, and 0.55 μg	Pandit et al., 2015
Orexin-A	Tocris Bioscience	0.0, 1.78, and 3.56 μg	Thorpe and Kotz, 2005; Zheng et al., 2007
Melanin concentrating hormone (MCH)	Tocris Bioscience	0.0, 0.5, and 1.0 μg	Georgescu et al., 2005
Corticotropin-releasing factor (CRF)	BACHEM	0, 50, and 250 ng	Peciña et al., 2006a; Chen et al., 2012
Urocortin (UCN)	BACHEM	0, 50, and 250 ng	

Each peptide was given at the doses listed, injected bilaterally in 0.5 μl of saline vehicle. On separate testing days, the same treatments were co-infused with 0.025 μg of the μ-opioid receptor agonist DAMGO (Tocris Bioscience). Doses were based upon prior work showing behavioral effectiveness after intracranial injections.

Across six separate drug test days, animals received intracranial injections of one of three doses (including the saline vehicle) of a single hypothalamic peptide alone or co-infused with 0.025 μg DAMGO. During vehicle and drug infusions, injection cannulas (30 gauge) were lowered bilaterally into the anterior medial nucleus accumbens and 0.5 μl of solution was delivered (at a rate of 0.32 μl per min) by a Harvard Apparatus (Holliston, MA, USA) microinfusion pump. Injectors remained in place for 1 min to allow for diffusion. After each drug administration, stylets were replaced and the animals were put into their feeding chamber for a 2-h session, during which food/water intake and movement was monitored.

Each rat received all possible drug combinations within their group, separated by at least 48-h to allow for washout of the drugs. Due to the high cost of the HRP agents, in most of the experiments, rats were tested first with all three doses of the hypothalamic peptide alone, in random order. In the last 3 days of the experiment, they received the same treatments in conjunction with 0.025 μg of DAMGO, once again with the order randomized. There was one exception: for Orexin-A treatments, all six possible treatments were given in random order for each rat across the 6 days of drug testing.

### 2.5 Histology

Once each experiment was completed, the rats were deeply anesthetized with sodium pentobarbital and perfused through the heart with a 0.9% buffered NaCl solution, followed by 10.0% formalin. Brains were removed and allowed to sink in 10.0% sucrose formalin before they were frozen and sliced into 60-μm sections with a cryostat. Sections were stained with cresyl violet. The tips of the cannulas were confirmed by light microscopy and charted with reference to Paxinos and Watson (1998). Only animals whose injectors were bilaterally placed within the medial nucleus accumbens were included in the behavioral analysis. All experiments began with 8 animals/group. Final *n*’s for each experiment are shown in the figure legends.

### 2.6 Data analysis

To determine if there were effects of drug treatment on food intake, repeated measures ANOVAs examined food intake across the session, with time (in 5-min bins), HRP treatment, and μ-opioid receptor stimulation as factors. Significant interactions were followed-up by conducting separate ANOVAs for the effect of HRP manipulation across time at both levels of DAMGO. Ambulation, rearing, and water intake were examined utilizing two-way repeated measures ANOVAs. *Post hoc* analyses were conducted on ambulatory measures when ANOVAs were significant, to determine the effects of DAMGO on the measures, as well as the effect of each individual peptide in the presence or absence of mu-opioid receptor stimulation.

## 3 Results

### 3.1 μ-Opioid receptor stimulation of the nucleus accumbens increases palatable eating

**FIGURE 2:**
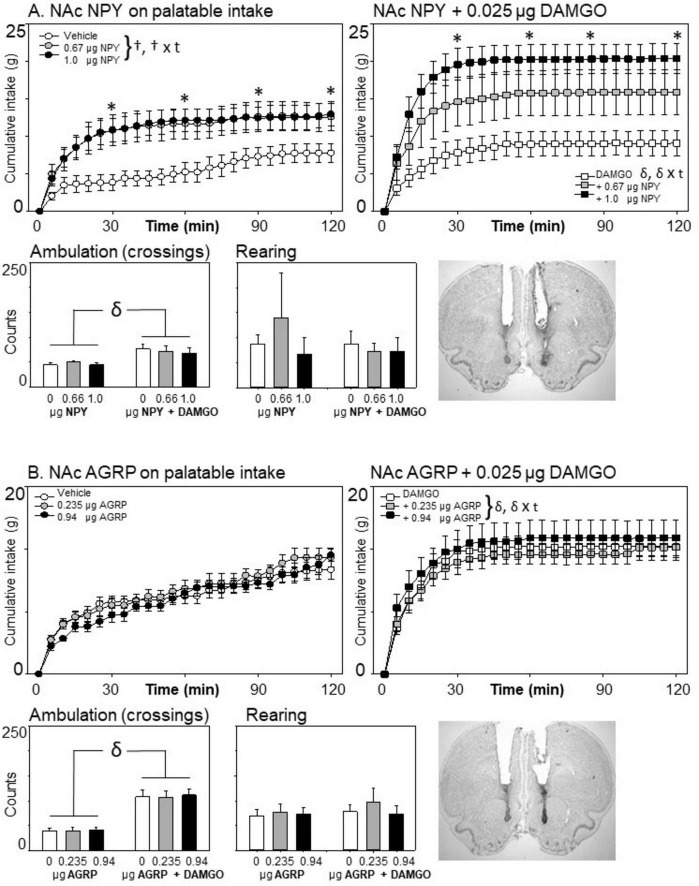
Effects of nucleus accumbens neuropeptide Y (NPY) (*n* = 8) or agouti-related protein (AGRP) (*n* = 8) and μ-opioid receptor stimulation on cumulative food intake and locomotion. Consistent with prior reports, DAMGO increased eating on the palatable diet across the 2-h session for these and all subsequent experiments (compare intake panels on left versus right for each experiment). **(A)** NPY injections increased food intake at both doses when injected alone, and significantly enhanced sweetened fat diet intake when co-injected with the μ-opioid receptor agonist DAMGO. **(B)** Although AGRP injections increased 24-h body weight (see text), it did not acutely affect palatable eating at the doses tested here, nor did it impact DAMGO-elicited eating. For all experiments, ambulatory activity was increased following μ-opioid receptor stimulation; neither NPY nor AGRP affected this increase. “†” indicates significant main effects of the tested homeostatic peptides (HRP) on the graphed behavior; “δ” indicates a significant main effect of mu-opioid receptor stimulation. “† × t” or “δ × t” indicate significant interactions between drug (the HRP or DAMGO) and time. Stars at 30, 60, 90, and 120 min reflect *post hoc* analysis of the HRP at those time points for the eating data. Each star signifies a significant effect of the HRP on eating as compared to the saline- (left) or DAMGO-only (right) condition. All data are represented as mean ± SEM. A representative photomicrograph showing injector placement is provided for each experiment.

**FIGURE 3:**
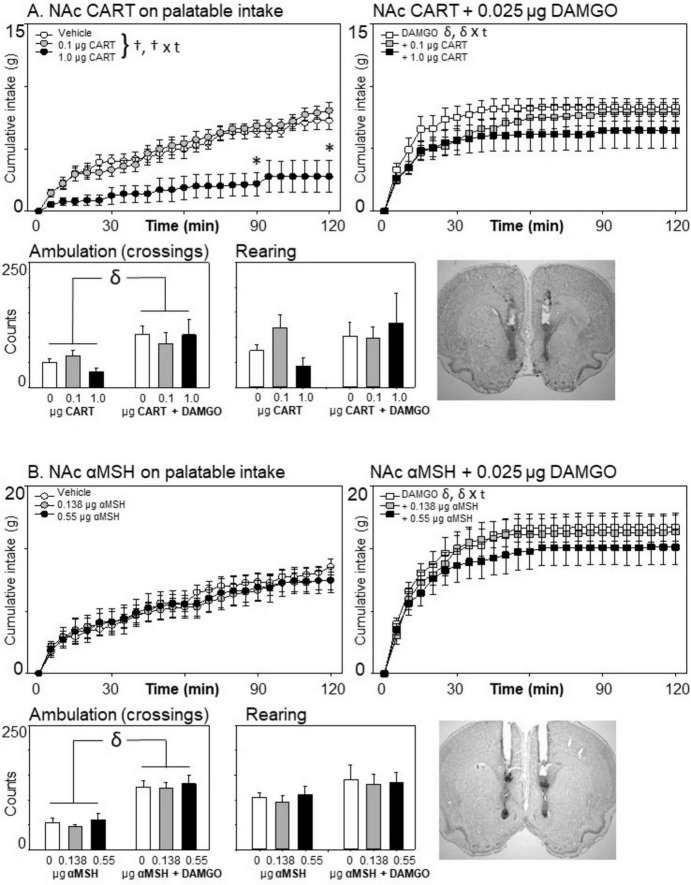
Effects of nucleus accumbens cocaine- and amphetamine-related transcript (CART) (*n* = 7) or alpha-melanocyte stimulating hormone (αMSH) (*n* = 8) and μ-opioid receptor stimulation on cumulative intake and locomotion. **(A)** At 1.0 μg/side, CART inhibited the intake of the sweetened fat diet, both on its own and when co-injected with DAMGO. **(B)** αMSH did not affect consumption, either on its own or in conjunction with μ-opioid receptor stimulation. Statistical symbols are as in Figure 2.

**FIGURE 4:**
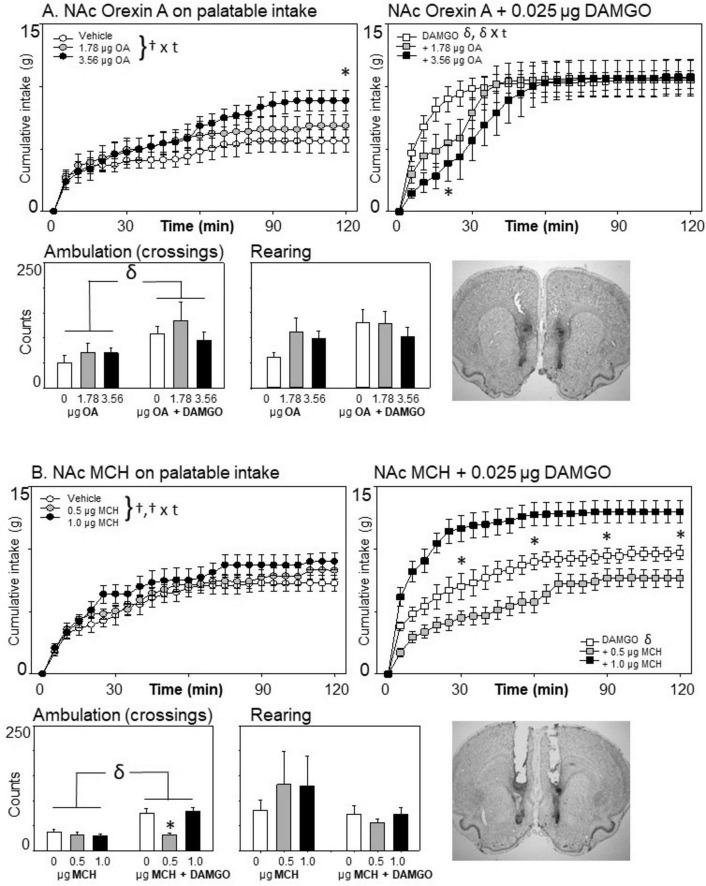
Effects of nucleus accumbens Orexin-A (*n* = 7) or melanin concentrating hormone (MCH) (*n* = 8) and μ-opioid receptor stimulation on cumulative intake and locomotion. **(A)** At 3.56 μg/side, Orexin-A modestly but significantly increased intake of the sweetened fat diet at 2 h. When injected with DAMGO, Orexin-A injections dose-dependently reduced and delayed the effect of μ-opioid receptor stimulation on intake, although the effects were overcome by the end of the 2-h session. **(B)** MCH did not affect intake on its own, but had differential effects at the two doses given, where 0.5 μg/side reduced DAMGO-elicited eating and the 1.0 μg/side dose enhanced it. The 0.5 μg/side dose also attenuated the ambulatory increase caused by μ-opioid receptor stimulation of the nucleus accumbens, as indicated by the star. Statistical symbols are as in Figure 2.

**FIGURE 5:**
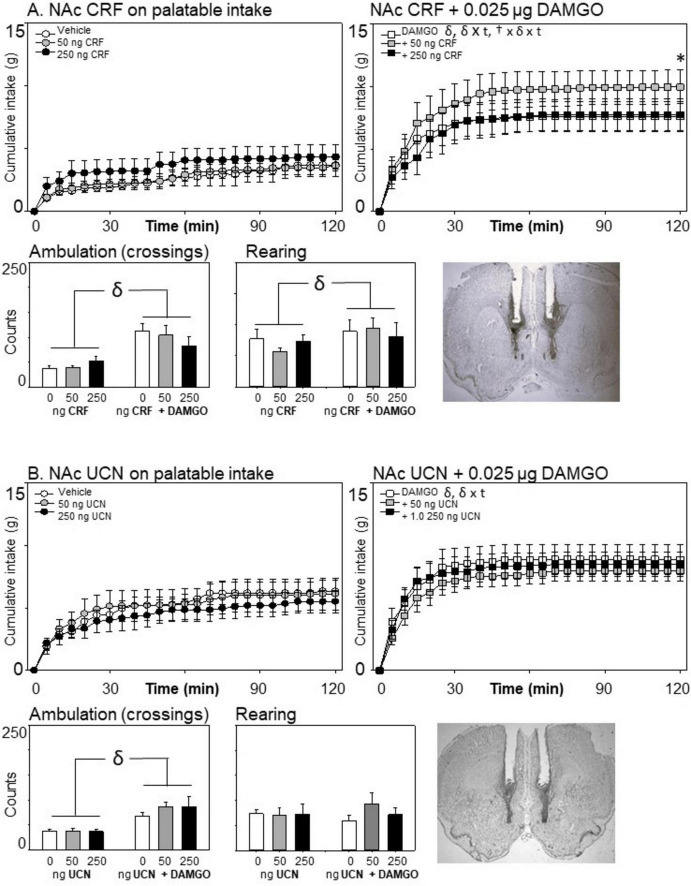
Effects of nucleus accumbens corticotropin-releasing factor (CRF) (*n* = 7) or urocortin (*n* = 8) and μ-opioid receptor stimulation on cumulative intake and locomotion. Neither stress-associated peptide affected eating on the palatable diet when injected alone. **(A)** A modest but significant increase in intake was observed 2 h after co-injection with the μ-opioid receptor agonist DAMGO for the 50 ng, but not the 250 ng, dose of CRF. **(B)** Urocortin had no effect on consumption or locomotion. Statistical symbols are as in Figure 2.

The effect of μ-opioid receptor stimulation of the nucleus accumbens on the intake of high-fat diets is well characterized in previous studies from multiple laboratories (e.g., Bakshi and Kelley, 1993; MacDonald et al., 2003; Peciña and Berridge, 2005; Zheng et al., 2010). Even at the relatively low threshold dose that we chose for these experiments (Parker et al., 2015), injections of DAMGO significantly increased overall food intake in each of the current eight experiments (all *p*’s < 0.05). There was also a significant DAMGO × time interaction effect for all the experiments but one (the exception being the MCH experiment). The effects of DAMGO on the sweetened fat diet intake can be observed in the figures for each experiment (Figures 2–5), and the relevant statistical verification of the eating increase is provided in Table 2. In addition to its effects on eating, DAMGO injections also significantly increased ambulation in the chamber in all experiments, as measured by end-to-end crossings. Water intake in the presence of the sweetened fat diet was generally low (< 4 grams per session), and none of the treatments given in these experiments yielded a significant main effect of the HRP treatment or DAMGO on water intake. Therefore, the remainder of this results section will focus on the impact of each HRP on consummatory and locomotor behavior, including their interactions with the known effects of μ-opioid receptor stimulation on palatable eating when relevant.

**TABLE 2:** Effects of DAMGO on palatable eating after injection into the anterior medial nucleus accumbens.

Experiment	DAMGO: main effect on eating	DAMGO × time interaction	DAMGO: ambulation effect
Exp 1 (NPY)	*F*(1,7) = 21.31 *p* = 0.002, η^2^ = 0.75	*F*(23,161) = 10.80 *p* < 0.001, $\eta_{p}^{2}$ = 0.61	*F*(1,7) = 6.00 *p* = 0.044, $\eta_{p}^{2}$ = 0.46
Exp 2 (AGRP)	*F*(1,7) = 10.92 *p* = 0.013, η^2^ = 0.61	*F*(23,161) = 14.02 *p* < 0.001, $\eta_{p}^{2}$ = 0.67	*F*(1,7) = 69.02 *p* < 0.001, $\eta_{p}^{2}$ = 0.91
Exp 3 (CART)	*F*(1,6) = 23.07 *p* = 0.003, η^2^ = 0.79	*F*(23,138) = 7.3 *p* < 0.001, $\eta_{p}^{2}$ = 0.55	*F*(1,6) = 11.1 *p* = 0.015, $\eta_{p}^{2}$ = 0.65
Exp 4 (αMSH)	*F*(1,7) = 14.626 *p* = 0.007, η^2^ = 0.68	*F*(23,161) = 5.824 *p* < 0.001, $\eta_{p}^{2}$ = 0.45	*F*(1,7) = 37.949 *p* < 0.001, $\eta_{p}^{2}$ = 0.84
Exp 5 (Orexin-A)	*F*(1,6) = 100.23 *p* < 0.001, $\eta_{p}^{2}$ = 0.94	*F*(23,128) = 7.49 *p* < 0.001, $\eta_{p}^{2}$ = 0.56	*F*(1,6) = 121.77 *p* < 0.001, $\eta_{p}^{2}$ = 0.95
Exp 6 (MCH)	*F*(1,7) = 8.83 *p* = 0.02, $\eta_{p}^{2}$ = 0.56	*F*(23,161) = 0.37 *p* = 0.999, $\eta_{p}^{2}$ = 0.04	*F*(1,7) = 43.66 *p* < 0.001, $\eta_{p}^{2}$ = 0.86
Exp 7 (CRF)	*F*(1,6) = 27.029 *p* = 0.002, $\eta_{p}^{2}$ = 0.82	*F*(23,138) = 11.26 *p* < 0.001, $\eta_{p}^{2}$ = 0.65	*F*(1,6) = 25.55 *p* = 0.002, $\eta_{p}^{2}$ = 0.81
Exp 8 (UCN)	*F*(1,7) = 10.50 *p* = 0.014, $\eta_{p}^{2}$ = 0.60	*F*(23,161) = 3.14 *p* < 0.001, $\eta_{p}^{2}$ = 0.31	*F*(1,7) = 44.30 *p* < 0.001, $\eta_{p}^{2}$ = 0.86

Consistent with past reports, μ-opioid receptor stimulation of the nucleus accumbens increased palatable eating and ambulation following DAMGO injections. This table summarizes these effects for each of the eight experiments presented here.

### 3.2 NPY or AGRP effects on palatable eating with and without μ-opioid receptor stimulation

#### 3.2.1 Neuropeptide Y

As can be seen in Figure 2A, injections of NPY into the anterior medial nucleus accumbens stimulation increased intake of the sweetened fat diet [Main effect of NPY: *F*(2,14) = 14.42, *p* < 0.001, η^2^ = 0.673; NPY × time interaction: *F*(46,32) = 9.39, *p* < 0.001, η^2^ = 0.400]. The interaction effect of NPY and DAMGO was also significant [*F*(2,14) = 3.88, *p* = 0.046, η^2^ = 0.357], although the three-way interaction of NPY, DAMGO, and time just missed significance [*F*(46,322) = 1.38, *p* = 0.062, η^2^ = 0.164]. This interaction is likely due to a synergistic effect of both agents on eating when combined (see Figure 2A, top right panel). To better characterize the effect of NPY when given alone versus with DAMGO, follow-up ANOVAs assessed the effect of NPY within each condition of DAMGO. NPY increased eating when given alone [*F*(2,14) = 14.74, *p* < 0.001, η^2^ = 0.678] and also when given with DAMGO [*F*(2,14) = 10.66, *p* = 0.002, η^2^ = 0.604]. To assess the time course of the changes in intake, *post hoc* assessments were made at 30, 60, 90, and 120 min, comparing each level of NPY to the vehicle condition during saline and DAMGO conditions using Fisher’s LSD. NPY significantly increased cumulative intake by 30 min into the session. Eating then leveled off, although total intake at the end of the session was still significantly greater when NPY was administered at the end of the 2-h session than when it was not present (following either saline or DAMGO infusion).

As has been regularly reported, μ-opioid receptor stimulation increased ambulatory activity [*F*(1,7) = 6.00, *p* = 0.044, η^2^ = 0.462], but did not affect rearing behavior in this experiment [*F*(1,7) = 0.37, *p* = 0.561, η^2^ = 0.051]. NPY did not affect cage crossing or rearing, nor did its administration interact with DAMGO’s effects on locomotor activity (all *p*’s > 0.25).

#### 3.2.2 Agouti-related protein

In contrast to the potent effects of NPY on sweetened fat diet intake, we observed no significant main effects of intra-accumbens AgRP on palatable consumption during the 2-h testing period [Figure 2B; Main effect of AGRP: *F*(2,14) = 0.10, *p* = 0.904, η^2^ = 0.014; AGRP × Time Interaction: *F*(46,322) = 0.95, *p* = 0.576, η^2^ = 0.119]. Co-infusion of AGRP did not affect the increased consumption caused by μ-opioid receptor stimulation, *F*(2,14) = 1.97, *p* = 0.177, η^2^ = 0.219, nor was there a significant three-way interaction effect between μ-opioid receptor stimulation, AGRP dose, and time, *F*(46,322) = 1.008, *p* = 0.464. μ-Opioid receptor stimulation increased ambulation [*F*(1,7) = 69.02, *p* < 0.001; η^2^ = 0.908] but not rearing [*F*(1,7) = 0.0.44, *p* = 0.526, η^2^ = 0.060]. AGRP injections did not affect locomotor measures (all main effects and interactions *p* > 0.10).

Although weight changes were not a primary dependent variable for these experiments, consistent with prior effects reported following ICV injections (Hagan et al., 2000), AGRP injections into the nucleus accumbens resulted in an obvious and significant gain in weight 24 h after the injections, *F*(2,14) = 7.89, *p* < 0.001, η^2^ = 0.712. Rats injected with vehicle lost −0.12 ± 1.23 g (mean ± SE), whereas the 0.235 μg/side of AGRP increased by 7.66 ± 2.71 g, and 0.94 μg/side AGRP increased by 20.37 ± 3.98 g.

### 3.3 The effects of CART and αMSH on palatable eating

#### 3.3.1 Cocaine- and amphetamine-related transcript

Consistent with all of these experiments, injections of 0.025 DAMGO into the nucleus accumbens increased consumption on the sweetened fat diet [*F*(1,6) = 23.07; *p* = 0.003, η^2^ = 0.794]. In contrast to the pro-eating effects of DAMGO, injections of 1.0 μg of CART reduced eating on the palatable diet [*F*(2,23) = 4.23, *p* = 0.023, η^2^ = 0.466; CART × time: *F*(46,276) = 3.93; *p* < 0.001; see Figure 3A]. However, the two drug treatments did not interact with each other on food intake [*F*(2,12) = 1.89, *p* = 0.193, η^2^ = 0.240], nor was there a three-way interaction with DAMGO, CART, and time of the session [*F*(26,276) = 1.20, *p* = 0.19, η^2^ = 0.167].

μ-Opioid receptor stimulation increased locomotion [*F*(1,6) = 11.1, *p* = 0.015, η^2^ = 0.649], but there was no effect of CART injection on ambulation, nor a DAMGO × CART interaction effect (all *p*’s > 0.10). Neither of the drugs had any effect on rearing behavior across the eating session (all *p*’s > 0.10).

A notable and significant decrease in weight occurred 24 h following the injection of 1.0 μg CART into the nucleus accumbens, *F*(2,12) = 11.98, *p* = 0.001, η^2^ = 0.666. Rats lost 7 ± 1.93 g of weight in the 24 h following this dose, whereas they gained 1.14 ± 0.72 g following vehicle injection and 1.71 ± 1.07 g following the 0.1 μg of CART.

#### 3.3.2 Alpha-melanocyte stimulating hormone

In contrast to the robust inhibition that was observed following CART injections into the anterior medial nucleus accumbens, we found no significant main effect of α-MSH injection on palatable food intake [*F*(2,14) = 0.873, *p* = 0.439, η^2^ = 0.111; α-MSH × time: *F*(46,322) = 0.673, *p* = 0.949, η^2^ = 0.088] (Figure 3B). There were no interactions between DAMGO and α-MSH treatment on eating [*F*(2,14) = 0.768, *p* = 0.482, η^2^ = 0.099], nor was there a significant three-way interaction effect between DAMGO, α-MSH, and time [*F*(46,322) = 0.784, *p* = 0.841, η^2^ = 0.101].

Alpha-melanocyte stimulating hormone injection into the nucleus accumbens shell did not produce any locomotor changes on its own, nor did it impact the increase in ambulation caused by μ-opioid receptor stimulation (all *p*’s > 0.1; Figure 3B).

### 3.4 The effects of Orexin-A and MCH on palatable eating

#### 3.4.1 Orexin-A

Consistent with each of these experiments, a significant increase in palatable eating was observed following nucleus accumbens μ-opioid receptor stimulation with DAMGO [*F*(1,6) = 100.256, *p* < 0.001, $\eta_{p}^{2}$ = 0.944; DAMGO × time: *F*(23,128) = 7.49, *p* < 0.001, $\eta_{p}^{2}$ = 0.555]. In the omnibus ANOVA, Orexin-A receptor stimulation yielded a significant Orexin-A × time interaction [*F*(46,276) = 5.84, *p* < 0.001, $\eta_{p}^{2}$ = 0.49], but not a significant main effect of Orexin-A (*F*(2,12) = 0.038, *p* = 0.963, $\eta_{p}^{2}$ = 0.006). The three-way interaction was not significant [*F*(46,276) = 0.944, *p* = 0.578,$\eta_{p}^{2}$ = 0.136]. However, given that the effects of Orexin-A treatment appeared to be different in the presence or absence of DAMGO (see Figure 4A), we conducted follow-up analyses to examine the impact of Orexin-A when given alone, and then again when given with DAMGO. Orexin-A significantly affected diet intake in both conditions, albeit in different ways. When given on its own, Orexin-A significantly increased food intake at the highest (3.57 μg) dose [Orexin-A × time interaction effect: *F*(46,276) = 3.90, *p* < 0.001, $\eta_{p}^{2}$ = 0.394], showing significantly increased eating (compared to vehicle injection) by 90 min and remaining until the end of the session. However, when Orexin-A was given with DAMGO, its effect was to initially disrupt the diet consumption early in the session [Orexin-A × time interaction: *F*(46,276) = 3.07, *p* < 0.001, $\eta_{p}^{2}$ = 0.338]. This initial disruption, however, was short-lived, as it was significant at 20 min into the session but not at 30 min, and there was no evidence of a change in DAMGO-elicited palatable diet intake due to Orexin-A by the end of the session.

μ-Opioid receptor stimulation had no significant effect on rearing [*F*(1,6) = 2.13, *p* = 0.20, $\eta_{p}^{2}$ = 0.26], but significantly increased ambulation [*F*(1,6) = 121.77, *p* < 0.001, $\eta_{p}^{2}$ = 0.95]. Orexin-A treatment did not impact ambulation or rearing, nor did it interact with DAMGO treatment (all *p*’s > 0.10).

#### 3.4.2 Melanin concentrating hormone

As can be seen in Figure 4B, MCH did not impact food intake when it was given alone, but demonstrated significant effects on the intake of the palatable diet when co-administered with the μ-opioid receptor agonist DAMGO [main effect of MCH: *F*(2,14) = 27.59, *p* < 0.001, $\eta_{p}^{2}$ = 0.798; MCH × time: *F*(46,322) = 2.32, *p* < 0.001, $\eta_{p}^{2}$ = 0.249; MCH × DAMGO: *F*(2,14) = 10.98, *p* = 0.001, $\eta_{p}^{2}$ = 0.249]. Surprisingly, the effect was biphasic, with co-injection of the lower 0.5 μg dose of MCH significantly reducing the intake of the sweetened fat diet compared to DAMGO treatment, and the higher 1.0 μg dose of MCH significantly increasing consumption above that of μ-opioid receptor stimulation.

In addition to the significant increase in ambulation caused by DAMGO [*F*(1,7) = 43.66, *p* < 0.001, $\eta_{p}^{2}$ = 0.249], MCH also affected crossings in the chamber [main effect of MCH: *F*(2,7) = 14.42, *p* < 0.001, $\eta_{p}^{2}$ = 0.673; DAMGO × MCH interaction: *F*(2,14) = 8.64, *p* = 0.004, $\eta_{p}^{2}$ = 0.552]. Follow-up analyses indicated that the effect of MCH on locomotion was significant only when DAMGO was present [*F*(2,14) = 14.15, *p* < 0.001, $\eta_{p}^{2}$ = 0.669]. Specifically, DAMGO-elicited ambulatory increases were inhibited by the 0.5 μg dose of MCH. Rears were unaffected by either MCH treatment or μ-opioid receptor stimulation.

### 3.5 The effects of CRF and urocortin on palatable eating

#### 3.5.1 Corticotropin-releasing factor

Although there was no main effect of CRF on eating [*F*(2,12) = 1.60, *p* = 0.210], there was a significant three-way interaction effect of DAMGO × CRF × Time [*F*(46,276) = 1.73, *p* = 0.004, $\eta_{p}^{2}$ = 0.224 (see Figure 5)]. To determine the cause of this interaction, we examined the effects of CRF on food intake separately for the DAMGO and non-DAMGO conditions. CRF did not significantly impact consumption in the absence of DAMGO, *F*(2,12) = 1.40, *p* = 0.284. However, when co-infused with DAMGO, CRF did significantly alter eating, *F*(2,12) = 4.18, *p* = 0.042, $\eta_{p}^{2}$ = 0.410, and there was a CRF by time interaction *F*(46,276) = 2.19, *p* < 001, $\eta_{p}^{2}$ = 0.268. At the end of the session, when rats received 50 ng of CRF in conjunction with DAMGO, there was a significant increase in eating in comparison to either DAMGO alone or DAMGO + 250 ng CRF. There were no impacts of CRF on either locomotion or rearing (*p*’s > 0.10). In this experiment, DAMGO increased rearing behavior, *F*(1,6) = 9.29, *p* = 0.023, $\eta_{p}^{2}$ = 0.608.

#### 3.5.2 Urocortin

Urocortin had no effects on eating [main effect of urocortin: *F*(2,14) = 0.701, *p* = 0.513; $\eta_{p}^{2}$ = 0.091; urocortin × Time: *F*(46,322) = 1.10, *p* = 0.31; $\eta_{p}^{2}$ = 0.136; urocortin × DAMGO × time: *F*(46,322) = 0.97, *p* = 0.531, $\eta_{p}^{2}$ = 0.122]. Likewise, it did not alter ambulation or rearing (all *p*’s > 0.10).

## 4 Discussion

The goal of these experiments was to broaden our understanding of the regulation of hedonic eating by hypothalamic HRP signaling within the nucleus accumbens. To our knowledge, this is the first comprehensive examination of all six of these HRPs (NPY, AGRP, CART, αMSH, Orexin-A, and MCH) utilizing identical behavioral procedures. We also examined whether the binge-like increase of food intake caused by nucleus accumbens injections of the μ-opioid receptor agonist DAMGO was modulated by co-injections of the hypothalamic HRPs. Finally, we sought to determine whether stress-related signaling (of CRF and urocortin) within the nucleus accumbens might impact eating in a similar manner.

### 4.1 HRPs and hedonic eating

Of the HRPs tested, only intra-accumbens injections of the orexigenic peptides NPY and Orexin-A significantly increased palatable eating by the end of the 2-h sessions when injected on their own. NPY fibers reach the nucleus accumbens via projections from the arcuate nucleus of the hypothalamus, in addition to other inputs (Pickel et al., 1998; Bagnol et al., 1999). Orexin projects to the region from neurons in the lateral hypothalamus (Urstadt and Stanley, 2015). These effects on eating are consistent with other reports that suggest that these peptides may increase consummatory motivational processes. Both NPY and Orexin-A increase eating following ICV administration (e.g., Levine and Morley, 1984; Sakurai et al., 1998). Furthermore, that NPY increased intake on a sweetened fat diet is consistent with prior work by Pandit et al. (2014), who reported increased intake of freely available sugar pellets after NPY injections into the nucleus accumbens. Similarly, Orexin-A injections increase food intake when injected into several brain regions (for a recent review see Mohammadkhani et al., 2024), and have been shown known to increase chow intake when injected into the nucleus accumbens (Thorpe and Kotz, 2005). Liu et al. (2020) recently demonstrated that Orexin-A injections into the nucleus accumbens increase intake of sweetened condensed milk (a palatable solution), and that blocking Orexin-A receptors there inhibits similar intake promoted by LHA stimulation. The effects of nucleus accumbens NPY and Orexin-A are likely not limited to consummatory processes, as there have been suggestions that may also influence appetitive, effortful processes (e.g., Pandit et al., 2013; Carney et al., 2023; Mohammadkhani et al., 2024).

Although the signaling actions of CART remain a mystery (for a recent review, see Ong and McNally, 2020), projections containing the peptide arise from the arcuate nucleus to innervate the nucleus accumbens and impact eating in food-restricted rats (Yang et al., 2005; Burghardt et al., 2016). Consistent with a role for CART in the homeostatic regulatory processes, our rats lost weight in the 24 h following treatment. Here, we showed for the first time that injections of CART into the nucleus accumbens also acutely reduces eating motivated by a palatable diet.

In contrast to the observed effects of NPY, Orexin-A, and CART on palatable eating, neither αMSH, AGRP, nor MCH impacted acute hedonic eating in these experiments when injected on their own. Notably, αMSH and AGRP have their actions at the same receptors that are implicated in homeostatic regulation of eating (MCR3 and 4), with the former acting as an agonist, and AGRP as an antagonist or inverse agonist (for a review, see Parker and Bloom, 2012). Previously, Pandit et al. (2015) reported that nucleus accumbens treatments of αMSH and AGRP decreased and increased appetitive motivation for sugar, respectively, as measured by an operant progressive ratio paradigm. However, when they examined free consumption of sugar pellets, the same treatments had no effect on consumption, consistent with what we report here. These results suggest that MCR4 receptors in the nucleus accumbens may preferentially impact effortful, appetitive motivation selectively, rather than affecting palatability in a manner that alters consummatory motivation.

Although we did not observe acute effects of AGRP on palatable eating, rats significantly gained weight in the 24 h following the 0.94 μg dose. This effect is consistent with other reports that have examined the longer-term effects of AGRP treatment on eating and body weight following intracranial injections, usually following intraventricular injection (e.g., Rossi et al., 1998; Small et al., 2001; Korner et al., 2003).

### 4.2 HRP effects on eating promoted by nucleus accumbens μ-opioid receptor stimulation

In addition to the effects of NPY, Orexin-A, and CART on hedonic eating, three of the orexigenic HRPs (NPY, Orexin-A, and MCH) and the anorexic HRP CART also impacted the binge-like eating elicited by nucleus accumbens μ-opioid stimulation with DAMGO. The patterns of those effects were remarkably different across peptides, suggesting unique interactions between μ-opioid signaling and each HRP within the nucleus accumbens. The most striking effect occurred following NPY administration. Co-administration of NPY and DAMGO increased palatable eating in a synergistic manner, suggesting that the two systems interact on a common output. Both μ-opioid receptor stimulation and NPY signaling in the nucleus accumbens can reduce neuronal communication (Yuan et al., 1992; van den Heuvel et al., 2015), and inhibition of neurons in the nucleus accumbens shell leads to voracious eating (Stratford and Kelley, 1997). It may be that activation of both signals lead to a more powerful inhibition of those neurons, leading to heightened intake by releasing downstream pathways. Although past studies have shown that intracranial or systemic opioid receptor blockade can attenuate NPY-induced intake (Rudski et al., 1996; Israel et al., 2005), and that Y1 receptor blockade can reduce μ-opioid elicited binging from within the nucleus accumbens (Zheng et al., 2010), the mechanism of the interactions of these systems within the nucleus accumbens remains to be determined.

The synergistic effects of NPY and DAMGO contrasted with the effects of co-administration of Orexin-A with μ-opioid receptor stimulation. In this case, Orexin-A delayed the pro-eating effects of DAMGO in the first 30 min of the session but did not subsequently affect the eating increase caused by DAMGO. This pattern of results suggests at least one of two potential explanations. First, Orexin-A signaling may directly interfere with the initial effectiveness of μ-opioid receptor signaling, an effect that dissipates across time. Second, the modest eating increase in eating caused by Orexin-A (when given alone) may have its effects through μ-opioid related signaling pathways, which are also engaged by DAMGO. These explanations are not mutually exclusive, and they are supported by previous work. For instance, the pro-eating effects of ICV injections of NPY, Orexin-A, and AGRP can be reduced by systemic pretreatment with an opioid antagonist (Kotz et al., 1995; Rossi et al., 1998; Clegg et al., 2002; Furudono et al., 2006). Of relevance to consumption promoted by a palatable diet, Furudono et al. (2006) showed that ICV injections of NPY, Orexin-A, and MCH all increased intake of a non-nutritive but palatable saccharin solution. When rats were pre-treated with naloxone, these increases were abolished (for Orexin-A) or attenuated (for NPY but not MCH). Additionally, Sweet et al. (2004) showed that opioid receptor blockade of the nucleus accumbens blocked Orexin-A-induced hyperphagia on rat chow, whether the Orexin-A was given into the lateral hypothalamus or the nucleus accumbens itself. Our data show for the first time that the nucleus accumbens may be an important node for the interaction of Orexin-A with opioid signaling in non-restricted rats motivated to eat by a palatable diet.

As noted above, MCH did not affect palatable eating when given on its own under these conditions. However, MCH injections into the accumbens had opposing behavioral effects on μ-opioid-induced eating dependent upon the dose used: at 0.5 μg palatable eating was inhibited, but the higher dose (1.0 μg/side) enhanced eating on the palatable diet, even though there were not significant effects of MCH when it was injected on its own. This demonstrates a concentration-dependent effect of MCH on opioid-promoted eating. These novel findings contrast with prior work (Lopez et al., 2011) demonstrating a modest increase in chow intake in lightly restricted rats with unilateral nucleus accumbens MCH. However, the authors also demonstrated that hedonic reactions to sweet solutions increased after nucleus accumbens injections of MCH, an effect that was abolished by opioid receptor blockade. Both our results and that of Lopez et al. (2011) suggest an interactive role of μ-opioid receptors and MCH on consummatory processes. Future research will be needed to further uncover the mechanisms for the interactions between MCH and opioid-receptor systems that underlie these effects.

Cocaine- and amphetamine-related transcript’s main effect on diet intake showed that its impact was due to a general suppression of eating both on its own and when co-infused with DAMGO, suggesting that mu-receptor stimulation and CART inhibition may work through separate mechanisms that sum together to direct eating.

### 4.3 Nucleus accumbens stress-related peptides and palatable eating

It was of interest to examine the potential effects of the stress-related peptides CRF and urocortin on palatable eating, since these signals are known to affect nucleus accumbens signaling, and in some cases have been shown to affect appetitive motivation for food- or drug seeking when injected into the nucleus accumbens. Stress can alter typical eating patterns, leading some individuals to engage in excessive intake of palatable foods—a behavior often referred to as emotional or comfort eating (Araiza and Lobel, 2018). Interestingly, individual differences play a role: stress increased palatable food intake in binge-prone rats, while binge-resistant rats showed no such effect (Calvez et al., 2016). Additionally, the CRF-urocortin family of stress-related peptides has been shown to reduce eating in other brain regions, including the hypothalamus, with urocortin often producing a more robust anorectic effect than CRF (Spina et al., 1996; Ohata et al., 2000).

Though CRF receptors are expressed in the nucleus accumbens and have been shown to modulate dopamine release and social behaviors (Lim et al., 2007; Lemos et al., 2012), their role in eating within this brain region is understudied. Some evidence suggests that CRF/urocortin signaling may impact food motivation through nucleus accumbens signaling, however. For instance, optogenetic stimulation of CRF-expressing neurons in the nucleus accumbens has been shown to amplify motivation to work for a sugary reward (sucrose), suggesting that these stress-related peptides may enhance craving for palatable foods (Baumgartner et al., 2021). Thus, we were interested how these signals might modulate consummatory motivation when rats were presented with a sweetened fat diet. Neither CRF nor urocortin affected palatable eating when injected on their own. This lack of an effect within the nucleus accumbens is interesting, given that arguments could be made that these stress-associated peptides might either reduce food-directed motivation by shifting motivational states away from resource-gathering (to promote protection of the organism) or toward increasing energy stores through increased eating in response to the presumed energy demands of a stress-induced state. Neither of these arguments are supported directly by our data, although there was a modest but significant facilitation of mu-opioid-elicited eating when the 50 ng dose (but not the 250 ng dose) of CRF was injected along with DAMGO. Regardless, these data provide additional evidence showing that the behavioral impact of nucleus accumbens pharmacological manipulations are selective to specific receptor classes and functions.

## 5 Conclusion: HRPs, the nucleus accumbens, and palatable eating

These data extend prior work examining the role of the nucleus accumbens in promoting food motivation in sated rats offered a palatable, sweetened-fat diet. Our findings support a role for the nucleus accumbens in promoting hedonic eating, but they also suggest that this form of intake remains sensitive to signals that arise from brain regions that have classically been considered as central for regulating eating in response to homeostatic need. That hedonic and homeostatic regulatory systems overlap and inform each other has been described by many (see section “1 Introduction”), but the nature of their interactions remain elusive, and the temptation to bifurcate their functions (and the brain regions which underlie them) remain. While the HRPs examined here are classically associated with hypothalamic control of energy balance (in addition to their other possible roles), their ability to modulate palatable food intake and interact with opioid-driven eating when injected into the nucleus accumbens demonstrates that these peptides also directly impact hedonic mechanisms that promote overconsumption directly at the level of mesolimbic circuits involved in action selection (e.g., Mogenson et al., 1980; Mizumori et al., 1999; Nicola, 2007; Floresco, 2015).

The distinct effects that we observed across the separate peptides, ranging from the synergic interactions of NPY with DAMGO to the dose-dependent modulation of opioid-elicited eating by MCH (and even CRF), indicate that these signals likely act through multiple, perhaps overlapping mechanisms within the nucleus accumbens that require additional study. CART’s suppressive effects, even in the presence of μ-opioid stimulation, further suggest that opposing hypothalamic influences converge on this region to shape palatable intake. For instance, it may be that these HRPs differentially impact the D1- or D2-expressing medium spiny neurons within the nucleus accumbens, which have recently been argued to play distinct roles in energy balance and food-motivated behavior (i.e., Zhu et al., 2016; Labouesse et al., 2018; Matikainen-Ankney et al., 2023). A better understanding of how these signals interact to influence the nucleus accumbens and other brain regions may serve us well as we seek to develop strategies and pharmacological approaches to better understand and treat maladaptive eating such as binging and obesity.

## Data Availability

The raw data supporting the conclusions of this article will be made available by the authors, without undue reservation.
